# Effect of Transcranial Direct Current Stimulation Combined with Donepezil on stroke patients with memory impairment

**DOI:** 10.12669/pjms.39.3.6822

**Published:** 2023

**Authors:** Wenqing Hu, Xue Wang, Xinyi Li, Qian Wang

**Affiliations:** 1 Wenqing Hu, Department of Rehabilitation, Tianjin Medical University General Hospital, Tianjin 300052, Tianjin, China; 2 Xue Wang, Department of Rehabilitation, Tianjin Medical University General Hospital, Tianjin 300052, Tianjin, China; 3 Xinyi Li, Department of Rehabilitation, Tianjin Medical University General Hospital, Tianjin 300052, Tianjin, China; 4 Qian Wang, Department of Rehabilitation, Tianjin Medical University General Hospital, Tianjin 300052, Tianjin, China

**Keywords:** Stroke, Memory impairment, Transcranial direct current stimulation, Donepezil

## Abstract

**Objective:**

To investigate the therapeutic effect of transcranial direct current stimulation (TDCS) combined with donepezil on stroke patients with memory impairment.

**Methods:**

The subjects of the study were 120 stroke patients with memory impairment admitted to the Rehabilitation Department of Tianjin Medical University General Hospital from July 2017 to March 2020. Enrolled patients were divided into Group-A (58 cases) and Group-B (62 cases) according to different treatment intervention methods. Patients in Group-A were treated with TDCS and those in Group-B received donepezil on the basis of TDCS. The changes in Montreal Cognitive Assessment (MoCA) memory index score, Barthel Index (MBI) score, cognitive function and cognitive potential were observed and compared between the two groups before and after treatment.

**Results:**

The improvement of total MoCA score, a single score of memory, MBI score, cognitive function and P300 potential index in Group-B was significantly better than that in Group-A (*p<*0.05).

**Conclusion:**

TDCS combined with donepezil can reduce or delay the cognitive impairment of stroke patients, improve their delayed memory ability, increase the neurotransmitter acetylcholine in the cerebral cortex, and further enhance their neural function. Findings in our study support that the proposed therapeutic method is worthy of clinical application.

## INTRODUCTION

Stroke, commonly known as “apoplexy”, has been the leading cause of disability in Chinese adults due to its incidence rate, mortality and disability rate. Memory dysfunction in stroke patients is mainly manifested in short-term memory impairment, such as time and spatial memory errors, decreased understanding ability, decreased learning ability, etc., which has a great impact on the quality of life and social adaptability of patients.^1^-^4^ According to previous research data^5^, in post-stroke disabled patients, 43.5% had cognitive impairment, while 45.4% had memory impairment. At present, there are some targeted drugs in clinical, yet with unsatisfactory therapeutic effects.

Transcranial direct current stimulation (TDCS) is a non-invasive brain stimulation technique that can directly stimulate cortical neurons. Meanwhile, donepezil can effectively improve the cognitive function of patients with Alzheimer’s disease and has a significant inhibitory effect on cerebral cholinesterase.^6^,^7^ Nevertheless, there are few studies on TDCS and donepezil in the treatment of stroke. Accordingly, the present study was performed with the discovery of good therapeutic effect by using TDCS combined with donepezil to treat 62 stroke patients with memory impairment.

## METHODS

The subjects of the study were 120 stroke patients with memory impairment treated in Tianjin Medical University General Hospital from June 2017 to March 2020. Enrolled patients were divided into two groups according to different treatment and intervention methods, including 58 cases in Group-A and 62 cases in Group-B. There were no significant differences in gender composition ratio, age and underlying diseases between the two groups (Table-I) (all *p*>0.05).This study was approved by the Institutional Ethics Committee of Tianjin Medical University General Hospital (No.: 2022013; Date: March 12, 2022), and written informed consent was obtained from all participants.

**Table-I:** Comparison of clinical conditions between the two groups.

Groups	n	Gender (n/n)	Age (years)	Duration of disease (Months)	Underlying disease (n/n)
	
		Male/Female			Hypertension/coronary heart disease/diabetes
Group-A	58	30/28	62.45±5.56	5.23±1.09	26/14/18
Group-B	62	35/27	62.34±5.72	4.98±0.97	31/16/15
Statistics	-	0.044	0.094	0.432	0.045
p value	-	0.834	0.926	0.897	0.823

### Inclusion criteria:

- Patients who met the diagnostic criteria in Semantics, Epidemiology and Semiology of Stroke (2018 Edition)^8^;
- Patients with memory impairment complained by the patient themselves or insiders;
- Patients with total score ≤26 points and memory score ≤4 points according to the Montreal Cognitive Assessment (MoCA) scale^9^;
- Patients without communication disorder;
- Patients and family members who knew and were willing to participate in the study.

### Exclusion criteria:

- Patients allergic to the studied drug;
- Patients with a history of mental illness;
- Patients taking memory-enhancing drugs;
- Patients with severe liver and kidney dysfunction.

Blood glucose, blood pressure, blood lipids and other indicators were controlled in all patients in both groups after enrollment. Besides, patients were provided targeted cerebral nerve protection. For example, patients with cerebral hemorrhage were given treatment such as prevention and treatment of cerebral edema and prevention of cerebral vasospasm, and patients with cerebral ischemia were given anti-platelet aggregation treatment. On this basis, patients in Group-A were treated with Donepezil Hydrochloride (Zhejiang Huahai Pharmaceutical Co., Ltd., National Medicine Permit No.: H20183417, specification of 5 mgx7 tablets x2 plates/box) orally (5 mg/time, once a day) for four weeks.

Patients in Group-B were given Donepezil Hydrochloride in the same dosage as above combined with TDCS. In terms of the therapeutic method of TDCS, patients were instructed to take a sitting position and use an electric stimulator (NeuroConn DC-stimulator plus; German). The coil diameter was set to 12 cm to stimulate the affected frontal lobe of patients. The peak stimulation intensity was 1.2 T, the pulse time was 100 s, and the stimulation frequency was 20 Hz. One sequence of stimulation was 30 times (5 d/week), and patients were treated for four weeks.

### Outcome measures:

*Memory score:* The seven indexes of attention and calculation, linguistic competence, abstract thinking, naming, delayed memory, and orientation were evaluated according to the MoCA test.^9^ Among them, the total MoCA score and the memory index score were considered the main indicators. The total score of the scale was 30 points, with normal cognitive function when the score was ≥26 points and normal memory when the score was ≥4 points according to the scoring criteria; *Activity ability:* The improved Barthel Index (MBI) scale^10^ was used to score patients, including 10 items of controlling urination, eating, dressing, going up and down stairs and walking. The total score was 100 points. Patients with higher scores indicated stronger daily activity ability based on the scoring criteria; *Cognitive function:* The Mini-Mental State Scale (MMSE)^11^ was used to evaluate 30 items of patients’ orientation, attention and calculation ability, memory ability, linguistic competence, etc. The total score was 30 points, and patients with a score ≥24 points were determined to be normal. Patients with higher scores had better cognitive ability; *Cognitive potential P300^12^*: P300 was used to detect the event-related potentials of patients. P300 amplitude and latency could effectively reflect the changes in patients’ psychological activities such as perception, comparison, memory, judgment and thinking before and after treatment.

### Statistical analysis:

Data processing used SPSS23.0 software. The measurement data was represented by (*χ̅*±*S*) and examined by the t-test, and the counting data was expressed as n (%) and detected by the *x*^2^ test. P*<*0.05 was used to indicate the existence of statistical differences.

## RESULTS

As shown in Table-II, the MoCA (total score and memory index score) and MBI scores of Group-B were higher than those of Group-A, with significant differences (all *p<*0.05). After treatment, the ability of orientation, attention and calculation, memory and linguistic competence in Group-B were higher than those in Group-A (all *p<*0.05). After treatment, P300 amplitude was longer and P300 latency was significantly shorter in both groups than those before treatment; meanwhile, the improvement of the P300 index in Group-B was significantly better than that in Group-A (all *p<*0.05; Table-IV).

**Table-II:** Comparison of MoCA and MBI scores between the two groups (*χ̅*±*S*, points).

Groups	Total MoCA score		MoCA-memory index score		MBI (points)	

	Before treatment	After treatment	Before treatment	After treatment	Before treatment	After treatment
Group-A (n=58)	15.74±5.48	18.12±4.26*	2.37±1.04	2.69±1.13*	24.81±7.40	35.32±5.35*
Group-B (n=62)	16.08±5.56	24.37±5.45*	2.41±1.05	3.50±1.12*	25.02±8.20	47.60±6.30*
*t*	0.114	7.629	1.059	7.284	0.719	10.593
p	0.909	0.000	0.292	0.000	0.474	0.000

***Note:***

* Compared with the score before treatment, p<0.05; MoCA: Montreal Cognitive Assessment scale; MBI: Improved Barthel Index.

**Table-III:** Comparison of cognitive function between the two groups (*χ̅*±*S*, points).

Groups	Orientation		Attention and calculation		Memory		Linguistic competence	

	Before treatment	After treatment	Before treatment	After treatment	Before treatment	After treatment	Before treatment	After treatment
Group-A (n=58)	2.29±0.48	5.62±1.50*	2.62±0.41	3.36±0.91*	2.07±0.37	3.62±0.65*	4.28±1.09	5.87±1.11*
Group-B (n=62)	2.31±0.49	6.43±1.49*	2.67±0.39	5.60±1.42*	2.10±0.41	6.25±1.43*	4.33±1.19	6.78±1.80*
t	0.116	2.966	0.105	7.180	0.451	8.432	0.210	3.306
p	0.908	0.004	0.917	0.000	0.734	0.000	0.834	0.001

***Note:***

* Compared with the score before treatment, p<0.05.

**Table-IV:** Comparison of P300 potential between the two groups (*χ̅*±*S*).

Groups	n	P300 amplitude (μV)		P300 latency (ms)	

		Before treatment	After treatment	Before treatment	After treatment
Group-A	58	6.42±1.14	8.65±1.79*	380.01±40.23	361.76±27.40*
Group-B	62	6.23±1.09	11.72±2.10*	378.27±41.74	321.70±24.29*
*t*	-	0.044	5.088	0.027	8.301
p	-	0.965	0.000	0.979	0.000

***Note:***

* Compared with the score before treatment, p<0.05.

## DISCUSSION

As reported by Orru G, Wang Z and Kucukdeveci AA et al.^13^-^15^, in the treatment of stroke, TDCS can effectively improve the distribution of degree in the left prefrontal lobe and left temporal lobe, shorten the path length of cerebral dysfunction, and improve the treatment efficiency. Accordingly, the present study analyzed the therapeutic effect of TDCS combined with Donepezil Hydrochloride on 62 stroke patients with memory impairment. In our study, the scores of MoCA, MBI and cognitive function were significantly higher in Group-B than those in Group-A (p<0.05), which were consistent with the above research results. Concerning the possible causes, TDCS has a unique effect on the hyperactivity changes of limb dyskinesia, aphasia, as well as hyperactivity of the spinal cord, nerve and other systems after stroke. It is a non-invasive technology to regulate the activity of cerebral cortex neurons through constant low-intensity DC current stimulation. It consists of two surface electrodes (positive and negative).

In the treatment of stroke, the weak polarized direct current is primarily used to regulate the activity of neurons in the cerebral cortex. At the neuronal level, the regulation of the excitability of the cerebral cortex by TDCS is mainly attributed to the polarity of stimulation.^16^,^17^Anodic stimulations can promote the excitation of the cerebral cortex, but the inhibition by cathodic stimulation is weakened. TDCS can not only affect cell membrane potential but also regulate synaptic plasticity by changing the activity of NMDA receptors and GABA.^18^

Furthermore, Donepezil Hydrochloride is a highly selective and reversible second-generation cholinesterase inhibitor. It can effectively improve the cognitive impairment of patients with Alzheimer’s disease and stroke. In terms of its functional mechanism, the drug can inhibit the degradation of acetylcholine (Ach), increase the content of acetylcholine, and improve the cognitive function and activity of daily life of patients.^19^ According to prior research carried out by Elsner B et al.^20^, TDCS can regulate cortical excitability and improve the clinical symptoms of post-stroke aphasia patients. The results of this study showed that the improvement of cognitive function and P300 potential indications in Group-B was better than that in Group-A (p<0.05), which was consistent with the above research. It suggests that combined use of TDCS and donepezil can improve cognitive ability, have a good effect on stimulating cerebral neuron activity, and can effectively enhance the memory ability of patients.

Stroke is cerebral ischemia caused by vascular stenosis or occlusion, and cerebral hemorrhage is caused by vascular rupture. Chronic cerebral dysfunction caused by long-term ischemia will lead to cognitive decline, including impairment in attention, memory, emotion, thinking, intelligence, etc.^21^-^24^ After the cerebral injury, the learning effect, memory ability and cognitive ability are reduced due to the damage to brain tissue structure or low perfusion and reduced metabolism.^25^,^26^

However, and importantly, the nervous system has plasticity to a certain extent, which can increase the dendritic branches of neurons under continuous external stimulation. Subsequently, there will be new axons and cell bodies that can facilitate the formation of new connections between neurons. Consequently, it can help to rebuild the neural network and brain function, so as to achieve the purpose of restoring cognition and memory.^27^,^28^

### Limitations:

However, this study was conducted in a single hospital with a small sample size. There is a need to further increase the sample size to improve the accuracy of results in our study.

## CONCLUSION

TDCS combined with donepezil can promote the recovery of cognitive function of stroke patients, improve their visuospatial skill and executive ability, enhance brain cell vitality, and promote the recovery of functional activities of cerebral cortex.

### Authors’ Contributions:

**WH** **and** **QW:** Designed this study, prepared this manuscript, are responsible and accountable for the accuracy and integrity of the work.

**XW:** Collected and analyzed clinical data.

**XL:** Data analysis, significantly revised this manuscript.
